# Treatment of ectopic variceal bleeding at choledochojejunostomy by endoscopic glue injection therapy with cyanoacrylate: Report of three cases including long‐term outcomes

**DOI:** 10.1002/deo2.110

**Published:** 2022-04-06

**Authors:** Tomohiro Tanikawa, Katsunori Ishii, Ryo Katsumata, Noriyo Urata, Ken Nishino, Mitsuhiko Suehiro, Miwa Kawanaka, Ken Haruma, Hirofumi Kawamoto

**Affiliations:** ^1^ Department of General Internal Medicine 2 Kawasaki Medical School Okayama Japan

**Keywords:** choledochostomy, cyanoacrylate, endoscopic glue injection therapy, pancreaticoduodenectomy, variceal bleeding

## Abstract

Ectopic varices around the choledochojejunostomy site after pancreatoduodenectomy are rare. Diagnosing ectopic varices is difficult but, if untreated or misdiagnosed, the resulting mortality is high. This report describes three cases of ectopic variceal bleeding at the choledochojejunostomy site that were improved by endoscopic glue injection therapy (EGIT) with cyanoacrylate (CA). Case 1 was a 68‐year‐old man admitted to the hospital with hematemesis and melena. Six years prior, the patient underwent a total pancreatectomy for intraductal papillary mucinous adenocarcinoma. We diagnosed ectopic variceal rupture at the choledochojejunostomy site and controlled bleeding by EGIT with alpha‐CA (αCA). Two recurrences of bleeding were improved by EGIT. Case 2 was a 71‐year‐old man admitted to the hospital with melena. Two and a half years prior, the patient underwent pancreatoduodenectomy for pancreatic head adenocarcinoma. We found the red plug on the ectopic varices at the choledochojejunostomy site through endoscopic observation and performed EGIT with αCA. He had no recurrence. Case 3 was a 77‐year‐old woman admitted to the hospital with melena. Eleven years prior, the patient underwent pancreatoduodenectomy for chronic pancreatitis at the pancreatic head. We controlled ectopic variceal bleeding at the choledochojejunostomy site by EGIT with αCA. Seven years after EGIT, ectopic varices could not be identified with an endoscope and there was no recurrence of ectopic bleeding.

## INTRODUCTION

The incidence of ectopic variceal bleeding not derived from esophagogastric varices is reported to be approximately 5% of all variceal bleeds. If untreated or misdiagnosed, the resulting mortality has been reported to be as high as 40%.^1^ Ectopic varices sometimes appear in the duodenum, jejunum, or rectum. Some previous reports have also reported ectopic variceal bleeding at the site of choledochojejunostomy anastomosis after pancreatoduodenectomy.^2^, ^3^ Ectopic varices form at the site of choledochojejunostomy due to portal venous obstruction and cavernous transformation of the portal vein.^4^ The major cause of portal venous obstruction is thrombosis or tumor involvement. After these events occur, collateral vessel formation and portal hypertension promote the formation of ectopic varices as hepatopetal collaterals. Although a usual jejunal varix can be seen with capsule endoscopy, it is impossible to observe choledochojejunostomy anastomosis due to its lack of passage.^5^ Therefore, diagnosing ectopic varices is difficult, especially varices at the site of choledochojejunostomy anastomosis with an afferent limb. Furthermore, even if we can detect intestinal bleeding due to ectopic variceal rupture, treatment of the bleeding is often difficult. In the previous reports, management of ectopic variceal bleeding was achieved by surgical therapy, endoscopic glue injection therapy (EGIT), portal venous stenting, and/or interventional radiology (IVR).^2^, ^3^, ^6^, ^7^ However, some reports of successful treatment with EGIT for ectopic variceal bleeding at choledochojejunostomy have only described the short‐term outcomes.^1^, ^3^, ^8^ Here, we report three cases in which EGIT with cyanoacrylate (CA) was successful in controlling ectopic variceal bleeding at the choledochojejunostomy site, including long‐term outcomes.

## CASE REPORT

### Case 1

**FIGURE 1:**
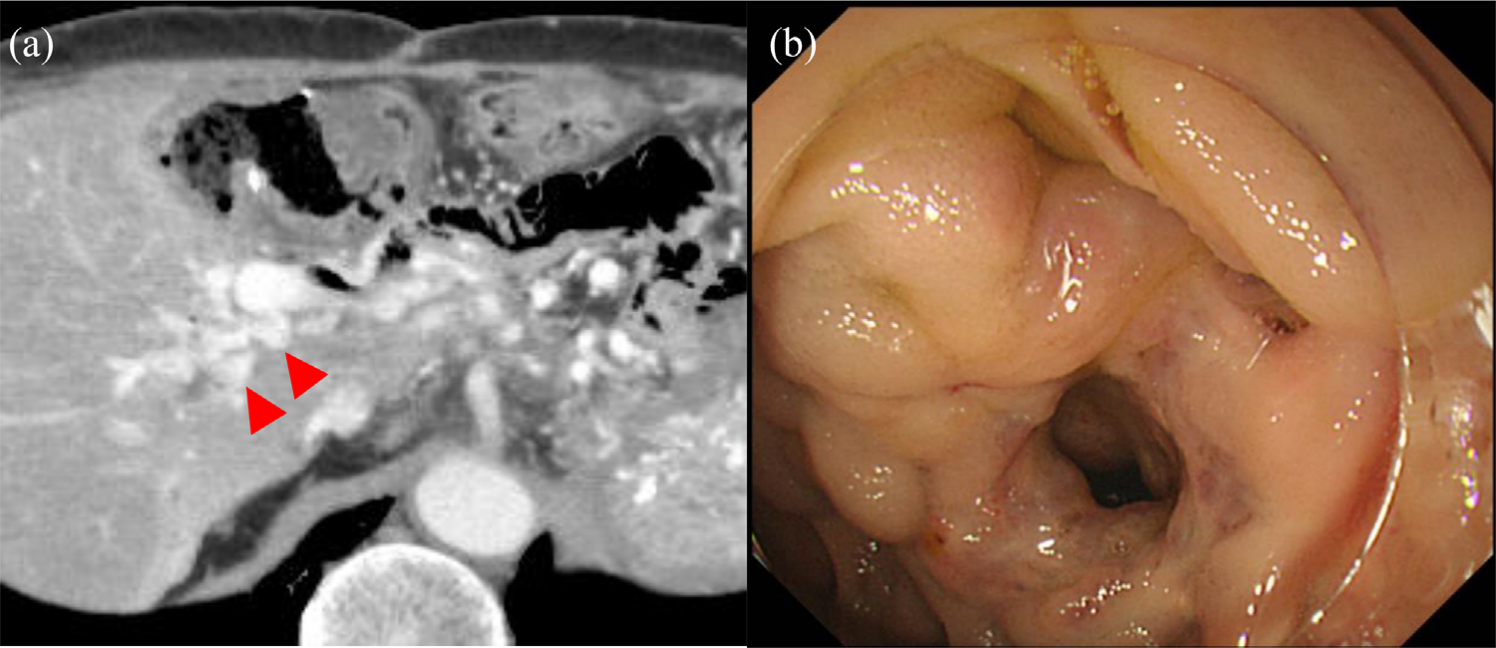
Images of case 1 before treatment: (a) Contrast‐enhanced computed tomography showed dilated veins around the choledochojejunostomy. (b) Ectopic varices around the choledochojejunostomy were observed by endoscopy

**FIGURE 2:**
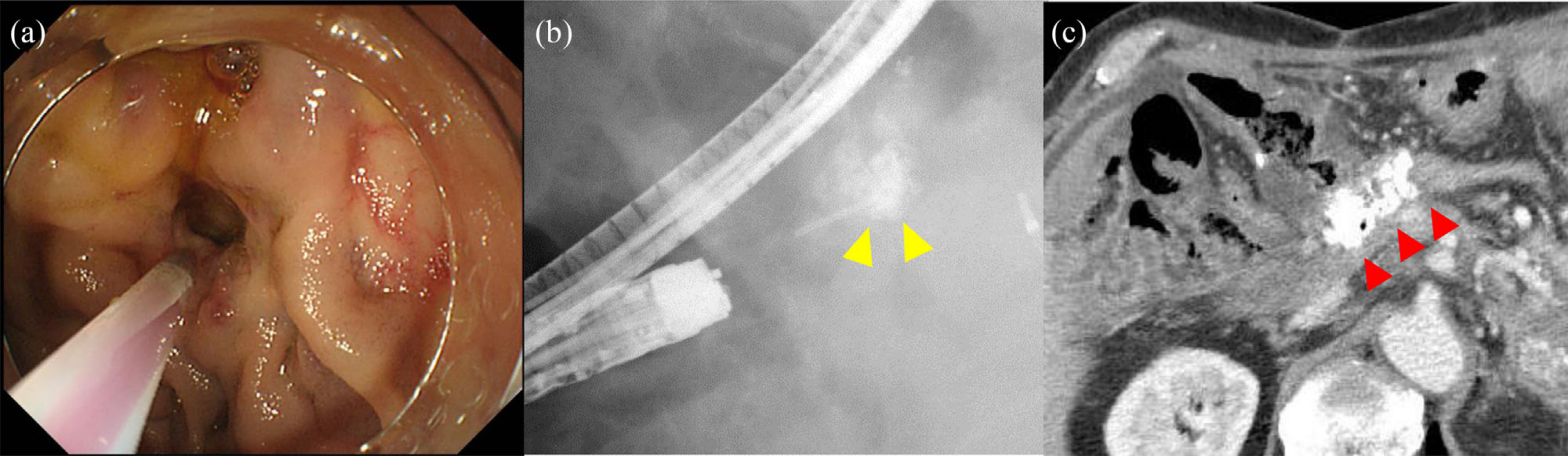
Endoscopic glue injection therapy of case 1: (a) We punctured varix with a 20‐gauge needle and recognized backflow. (b) We injected the cyanoacrylate (CA) with iodized poppy oil into varix. (c) Computed tomography after the procedure showed that the CA was retained in the ectopic varices around the choledochojejunostomy

A 68‐year‐old man was admitted to our hospital with hematemesis and melena. Six years prior, the patient underwent total pancreatectomy and choledochojejunostomy for intraductal papillary mucinous adenocarcinoma. Contrast‐enhanced computed tomography (CT) disclosed portal vein obstruction due to thrombosis and significantly dilated venous collaterals around the choledochojejunostomy anastomosis (Figure 1a). Since we could not find any abnormal findings through the conventional esophagogastroscopy, we suspected a possible ectopic variceal rupture around the choledochojejunostomy anastomosis. When the normal upper endoscope (GIF‐Q260J; Olympus Corporation, Tokyo, Japan) was advanced to the site of the choledochojejunostomy, we found ectopic varices on the mucosa around the anastomosis (Figure 1b) and considered that it was the reason for the bleeding. Puncturing ectopic varix with a 20‐gauge needle (Endoscopic Puncture Needle; TOP, Tokyo, Japan), we confirmed the backflow of blood, and then we injected 2.2 ml alpha‐CA (αCA) with 0.4 ml iodized poppy oil into varix (Figure 2a,b). We ensured there was no bleeding after the withdrawal of the needle and confirmed the completion of the procedure. CT after EGIT showed that the CA was retained in ectopic varix at the choledochojejunostomy despite sufficient portal venous flow into the liver (Figure 2c). Liver failure or liver dysfunction did not occur. Recurrence of ectopic variceal rupture occurred 6 months and 1 year after the first therapy. Both recurrences were treated with the same procedures. Approximately 1 year after the third therapy, ectopic variceal bleeding has not recurred.

### Case 2

**FIGURE 3:**
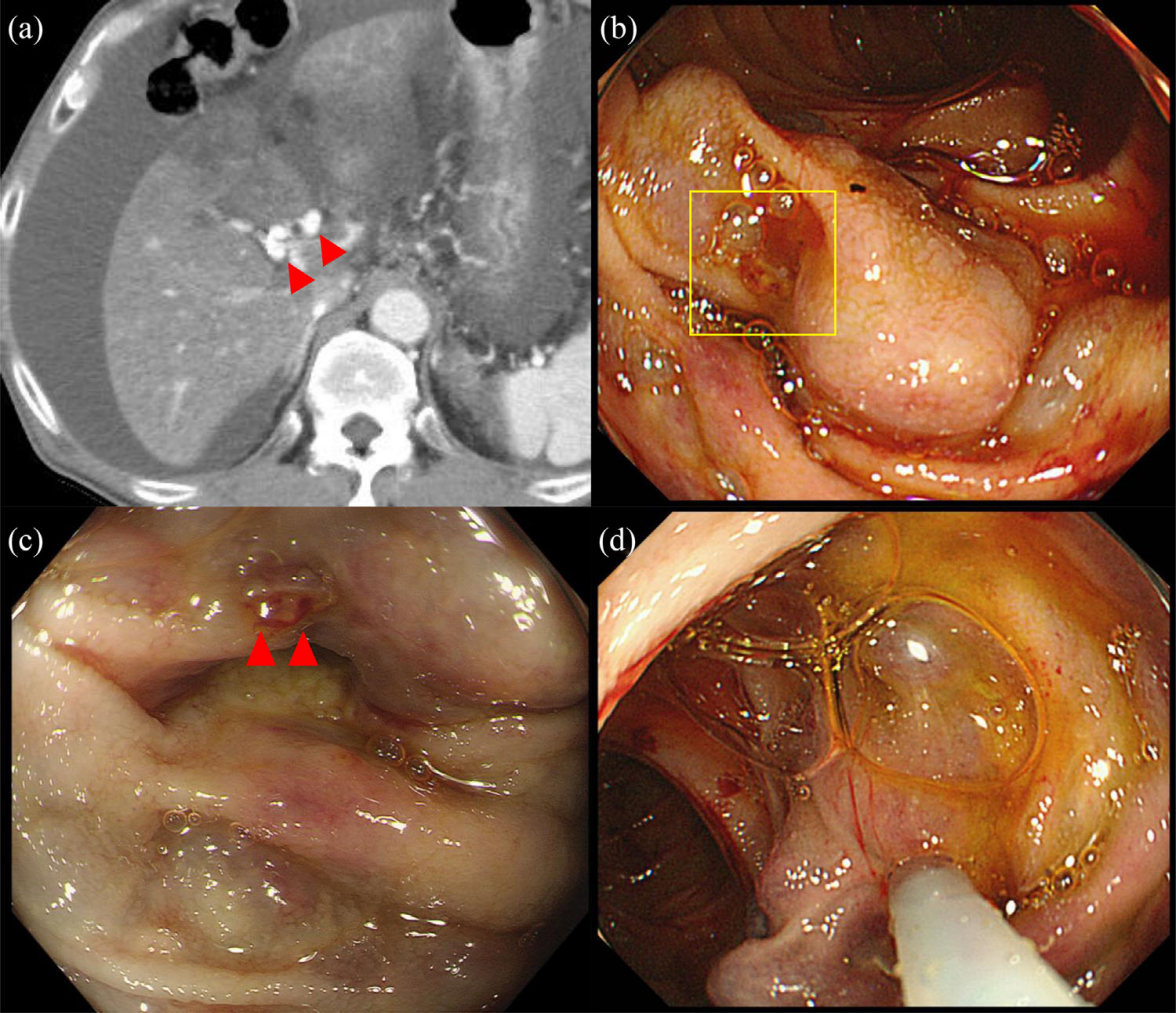
Images of case 2: (a) Contrast‐enhanced computed tomography showed dilated veins around the choledochojejunostomy. (b) Ectopic varices at the choledochojejunostomy were observed by endoscopy. (c) We found a red plug on the ectopic varix. (d) We punctured varix with a 20‐gauge needle and recognized backflow before injecting the cyanoacrylate with iodized poppy oil into the varix

A 71‐year‐old man was admitted to our hospital with melena. The patient underwent pancreatoduodenectomy and choledochojejunostomy for pancreatic head adenocarcinoma 2.5 years prior to this presentation. Eighteen months after the surgical therapy, the tumor had recurred. Although the patient received chemotherapy, the tumor progressed, and he finally received the best supportive care. He had anemia (hemoglobin 7.0 g/dl), portal vein obstruction via tumor embolism, and significant venous dilatation around the choledochojejunostomy anastomosis on contrast‐enhanced CT (Figure 3a). Without hemorrhagic findings in the esophagus and stomach, we judged that the bleeding was from ectopic varix rupture through the red plug (Figure 3b,c). We punctured ectopic varix with a 20‐gauge needle and injected 2.2 ml αCA with 0.4 ml iodized poppy oil into varix (Figure 3d). Two months after EGIT, the patient died from tumor progression without recurrence of variceal rupture.

### Case 3

**FIGURE 4:**
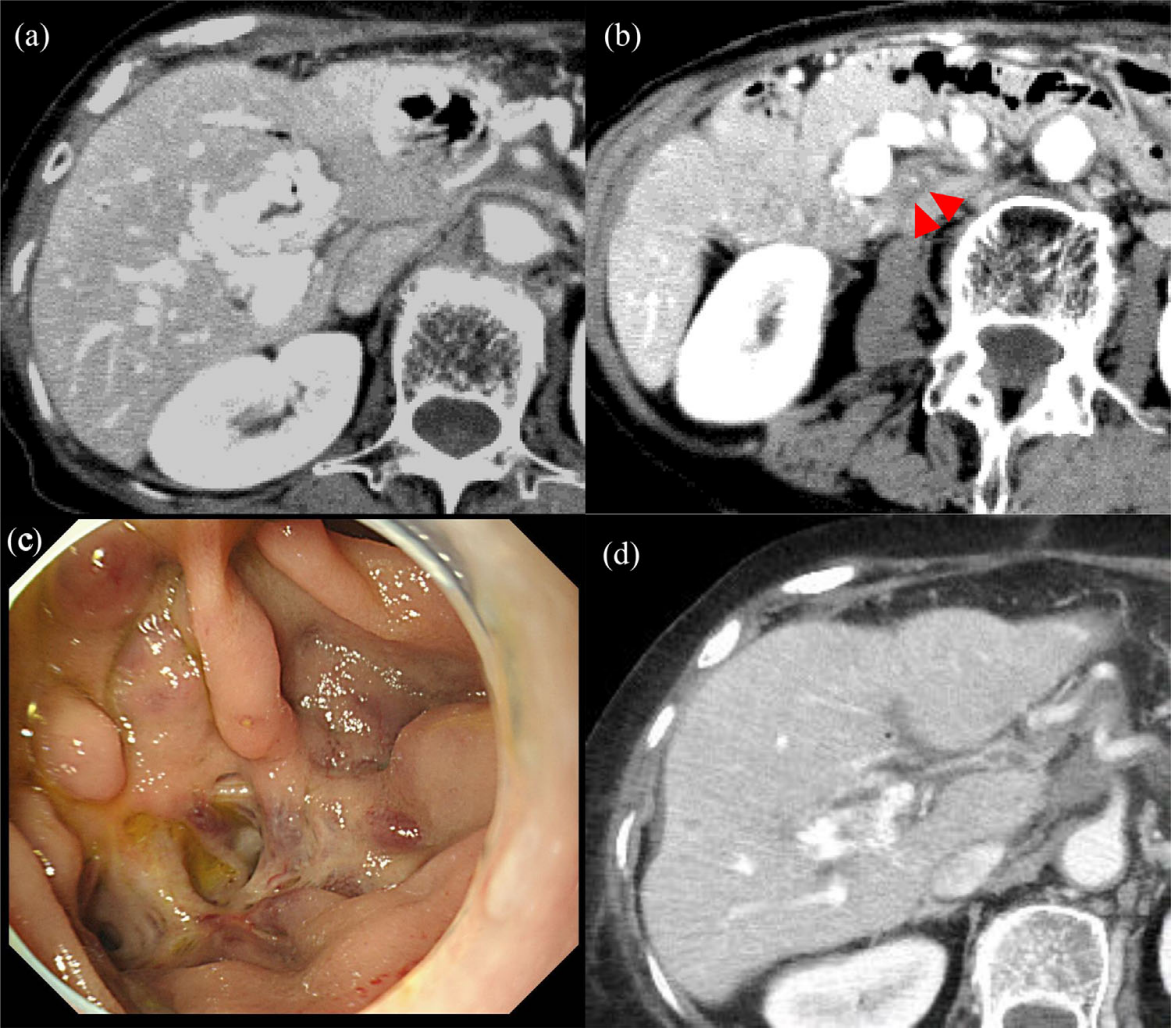
Images of case 3: (a, b) Contrast‐enhanced computed tomography (CT) showed dilated veins around the choledochojejunostomy and portal vein obstruction. (c) We found ectopic varices around the choledochojejunostomy due to portal vein obstruction. (d) CT performed 7 years after endoscopic glue injection therapy demonstrated fewer dilated veins around the choledochojejunostomy

A 77‐year‐old woman was admitted to our hospital with melena. Eleven years prior, the patient underwent pancreatoduodenectomy and choledochojejunostomy for chronic pancreatitis at the pancreatic head. Two years after surgery, melena was sometimes recognized; therefore, a survey for melena was performed at another hospital. However, the source of bleeding could not be identified through conventional endoscopy and capsule endoscopy. On contrast‐enhanced CT, we detected significantly dilated veins around the choledochojejunostomy anastomosis and portal vein obstruction by thrombosis (Figure 4a,b). To identify the source of the bleeding, we observed the choledochojejunostomy anastomosis using a normal upper endoscope and found ectopic varix (Figure 4c). Although we did not observe active bleeding or red plugs, there were no other possible sources of the bleeding. We performed EGIT on ectopic varix and injected 2.2 ml αCA with 0.4 ml iodized poppy oil using a 20‐gauge needle. Seven years after the previous therapy, CT showed that the significantly dilated ectopic varix at the choledochojejunostomy site was improved, and melena did not recur. In addition, ectopic varix around the anastomosis could not be identified using an endoscope (Figure 4d).

## DISCUSSION

In this report, we described three cases of ectopic variceal bleeding from choledochojejunostomy that were managed by EGIT with CA. CA and ethanolamine oleate (EO) are often employed in endoscopic injection sclerotherapy for esophagogastric varix. CA, which is a liquid tissue adhesive, is mainly employed for the treatment of gastric variceal bleeding. Upon the injection of CA into varices, it forms a polymer that instantly makes thrombi an embolic substance. Therefore, CA is effective in the hemostasis of varices with rapid blood flow. In contrast, EO is a detergent, which leads to an inflammatory reaction to the venous wall. As a result, thrombosis and possible occlusion of the vein are attained 3–5 min after its injection. Therefore, EO is not suitable for endoscopic treatment of varices with rapid blood flow. In all three cases, we employed CA as a hemostatic material. Injecting CA around the bleeding point on the varices cannot shut off the hepatopetal flow completely because this procedure can only attain local thrombosis in the varices. Although there has been a report of liver failure as a result of hemostasis,^9^ no liver failure and no diffuse spread of CA in the whole liver was found in any of our cases. In general, complications of EGIT with CA at the choledochojejunostomy site can include thrombosis, infection, bile duct stenosis, and acute bleeding. We did not experience any of these in our cases.

In two cases, recurrence of variceal bleeding (detected as melena) did not occur. In case 3, the ectopic varices at the choledochojejunostomy site almost disappeared 7 years after EGIT. Similar to gastric varix, ectopic varix at the choledochojejunostomy site could be improved by this EGIT in the long term. On the other hand, there were two recurrences of ectopic variceal bleeding in case 1 and they underwent EGIT with CA three times. EGIT with CA can not improve portal hypertension itself, and it needs to be reduced to prevent recurrence of variceal bleeding in this case. Sato et al. reported a case that required IVR after controlling bleeding with surgical ligation.^10^ Portal decompression therapy should be performed due to portal venous stenting or embolization with IVR. Saeki et al. reported that portal decompression therapy decreased rebleeding less than the obliteration of varix.^2^ Due to immediate hemostasis and sustained effectiveness, EGIT with CA can be a curative therapy in some cases. Therefore, when gastrointestinal bleeding occurs after choledochojejunostomy, we recommend endoscopic observation including choledochojejunostomy to identify the bleeding site. If the cause of bleeding is ectopic variceal rupture at the choledochojejunostomy site, we suggest EGIT with CA to control the bleeding and then other elective therapies to prevent recurrent bleeding.

## CONFLICT OF INTEREST

The authors declare that they have no conflict of interest.

## FUNDING INFORMATION

None.
